# Characterization of Metabolically Healthy Obese People and Metabolically Unhealthy Normal-Weight People in a General Population Cohort of the ABCD Study

**DOI:** 10.1155/2017/9294038

**Published:** 2017-08-03

**Authors:** Silvio Buscemi, Pierfilippo Chiarello, Carola Buscemi, Davide Corleo, Maria Fatima Massenti, Anna Maria Barile, Giuseppe Rosafio, Vincenza Maniaci, Valentina Settipani, Loretta Cosentino, Carla Giordano

**Affiliations:** ^1^Dipartimento Biomedico di Medicina Interna e Specialistica (DIBIMIS), University of Palermo, Palermo, Italy; ^2^Unit of Malattie Endocrine del Ricambio e della Nutrizione, AOU Policlinico “P. Giaccone”, Palermo, Italy; ^3^Dipartimento di Scienze per la Promozione della Salute e Materno Infantile, University of Palermo, Palermo, Italy

## Abstract

There is actually no consensus about the possibility that in some instances, obesity may be a benign metabolically healthy (MH) condition as opposed to a normal-weight but metabolically unhealthy (MUH) state. The aim of this study was to characterize MH condition and to investigate possible associations with metabolic and cardiovascular complications. One thousand nineteen people (range of age 18–90 years) of the cohort of the ABCD_2 study were investigated. Participants were classified as normal weight (BMI < 24.9 kg/m^2^) or overweight-obese (BMI ≥25 kg/m^2^); they were also classified as MH in the presence of 0-1 among the following conditions: (a) prediabetes/type 2 diabetes, (b) hypertension, (c) hypertriglyceridemia or low HDL cholesterolemia, and (d) hypercholesterolemia. MUH condition was diagnosed if ≥2 of the conditions listed were found. The prevalence of overweight/obese people was 71.1%, of whom 27.4% were found to be MH. In addition, 36.7% of the normal-weight participants were MUH. HOMA-IR, high sensitivity C-reactive protein, and the carotid intima-media thickness were significantly different in the 4 subgroups (*P* < 0.001), with higher values observed in the MUH normal-weight and obese groups. In conclusion, this study highlights the importance of identifying a MH condition in normal-weight and in obese people in order to offer better treatment.

## 1. Introduction

Obesity is a widespread condition in the Western world [1], and its prevalence is continuously increasing, even in emerging countries [2]. Parallel to obesity, the prevalence of type 2 diabetes (T2D), hypertension, and atherosclerotic cardiovascular disease is rising [3]. It has not been definitively established to what extent obesity is responsible for the epidemic of metabolic and cardiovascular comorbidities or if these conditions are more directly the consequence of unfavorable changes that deteriorate people's lifestyle in terms of sedentary habits and poor diet [4–6]. In fact, given the interaction between genotype and lifestyle, it has been hypothesized that obesity is not always uniquely responsible for classical comorbidities such as diabetes and atherosclerosis and that cases of uncomplicated obesity are not rare [7]. This point of view is of interest because, otherwise, we would characterize more than half of the adult population as sick.

It has therefore been proposed to classify obese people as metabolically healthy (MH) and unhealthy (MUH) [8]. Interestingly, as diabetes and atherosclerosis can occur even in normal-weight people, the MH and MUH conditions have also been applied to normal-weight people. Therefore, it is not rare to find normal-weight MUH individuals who have a worse prognosis than MH-O individuals [9]. This approach would be of particular usefulness in personalizing treatment and selecting subclasses of people in whom to potentiate interventions. Unfortunately, there is no consensus on the definition of MH and MUH conditions, and general population-based studies are scarce [10–12].

In this study, we investigated the association between MH and MUH conditions, defined on the basis of the components of metabolic syndrome and of blood cholesterol concentrations, with different cardiovascular and metabolic outcomes in a comprehensive general population cohort who participated in the second evaluation, in 2015, of the nutrition, cardiovascular wellness and diabetes (ABCD_2) study [13]. The aim was to characterize MH condition with respect to body size and investigate possible associations with factors predisposing to metabolic and cardiovascular complications.

## 2. Materials and Methods

The ABCD_2 project (ISRCTN15840340) is a longitudinal observational single-center study of a cohort representative of the general population living in Palermo, the largest city in Sicily, Italy, with a population of 674742. The ABCD study cohort was recruited in 2011, as previously described [13]. The demographic characteristics of the ABCD cohort were similar, even if nonoverlapping, to those of the general population of the same range of age (18–90 years) living in Palermo in 2011, as presented elsewhere [14] (Table 1). The original cohort was recontacted (telephone, e-mail, and letter) in 2015, and those who agreed to participate in the study were asked to come to the Metabolism and Clinical Nutrition Laboratory of the Department of Internal and Specialized Medicine at the University of Palermo and were reexamined from March 21 to July 31. Each participant was given the opportunity to invite a relative or friend as a new participant in the study, but the number of new participants was limited to the first 300 people. The demographic characteristics of the ABCD_2 cohort were not significantly different from those of the ABCD_1 cohort, as presented in Table 1.

Our institutional Ethics Committee (“Palermo 1” of the Policlinico “P. Giaccone” University Hospital, November 03, 2014, ref: 3/2015) approved the study protocol, and each participant signed an approved informed consent form.

Participants were administered a questionnaire on demographic characteristics, the presence of chronic diseases, and pharmacologic treatment. In particular, a specific questionnaire was developed for defining the individual level of habitual physical activity (HPA). The physical activity questionnaire consisted of the following 4 questions concerning the type and the frequency of exercise: (a) do you regularly engage in structured physical exercise (including walks of at least 20 minutes)?; (b) if yes, what kind of physical exercise?; (c) how many times a week?; and (d) what is your job? Thus, on the basis of both intensity and frequency of physical exercise and considering participants doing physically demanding jobs, 4 different levels of HPA were defined: (a) very low (no habitual exercise), (b) low (walks of 20–30 minutes at least 3 times a week); (c) medium (scheduled physical activity 1–3 times a week or walks > 30 minutes at least 3 times a week), and (d) high (scheduled physical activity > 3 times a week or competitive sport activity or heavy-job activity). The list of heavy-job activities is reported in Table 2. Furthermore, housewives under the age of 65, with children, were considered at a medium HPA or higher if the criteria were met for defining high HPA. We validated the HPA questionnaire in comparison (26 external participants, 7 females and 19 males, BMI: 31.0 ± 10.3 kg/m^2^) with 7-day pedometer measurements (nonobese: 5986 ± 1793 versus obese: 5328 ± 3027 steps/day; *P* = 0.54), obtaining significant correlations between the two methods (*r* = 0.57; *P* = 0.003).

Half-quantitative habitual intakes of different foods during the past 12 months were assessed using a food frequency questionnaire (FFQ) validated for the local population [15].

Participants underwent blood sampling for assessment of blood chemistry and hormonal parameters. For each participant, a blood sample was frozen and stored at −80°C for subsequent measurements. T2D and prediabetes were defined according to the most recent consensus statements [16]. Arterial hypertension was defined as systolic blood pressure values ≥ 130 mmHg or diastolic blood pressure values ≥ 85 mmHg or antihypertensive medication use; triglyceride concentrations ≥ 150 mg/dl or lipid-lowering medication use defined hypertriglyceridemia. Low HDL cholesterol levels were defined if <40 mg/dl for men and <50 mg/dl for women [17]. Hypercholesterolemia was defined as values > 200 mg/dl or use of cholesterol-lowering medication [18]. Participants were classified as MH if they had 0-1 conditions of the following: prediabetes/T2D, hypertension, hypertriglyceridemia or low HDL cholesterolemia, and hypercholesterolemia. Participants were defined as MUH if they had at least 2 of the conditions listed above.

Height and body weight were measured with participants lightly dressed and without shoes (SECA); the body mass index (BMI) was calculated as body weight (kg)/height^2^ (m^2^). Normal-weight (N) participants were defined if BMI values < 25 kg/m^2^; overweight/obese (O) people were defined if BMI values ≥ 25 kg/m^2^ [19]. Body circumferences were measured at the umbilicus (waist circumference) and at the most prominent buttock level (hip circumference); the waist-to-hip ratio (WHR) was used as an indirect index of body fat distribution. Systolic and diastolic arterial blood pressure (two measurements obtained at 5-minute intervals in seated position) and heart rate (Omron M6; Omron Healthcare Co., Matsusaka, Mie, Japan) were measured by physicians or dietitians according to standardized procedures. Body composition in terms of fat mass (FM) and fat-free mass (FFM) was estimated using bioelectrical impedance analysis (BIA; BIA-103; RJL, Detroit, USA/Akern, Florence, Italy) following the manufacturer's equations, as previously described [20].

The ankle-brachial index (ABI) was calculated as a measure of peripheral arterial obstructive disease and as a biomarker associated with mortality risk according to recent guidelines [21, 22]. Bilateral arm and ankle blood pressure values were measured, with the participant supine, with an automated oscillometric device that used a wireless and computerized system (iHealth Pro; iHealth Labs Europe, Paris, France). The ABI was calculated as the ratio of ankle-to-brachial systolic pressure. The worst ABI value of the 2 sides was considered for calculations. Participants were defined as having normal ABI measures if between 0.90 and 1.40.

Images of the right and left extracranial carotid artery walls were obtained in several projections with a high-resolution ultrasonographic 10 MHz linear array probe (Sonoline G50; Siemens, Germany). When an optimal longitudinal image was obtained, it was frozen and stored on digital support for subsequent off-line measurements of intima-media thickness (c-IMT). The end-diastolic c-IMT of the far wall of both common carotid arteries was measured as described elsewhere [23] and according to current guidelines [24]. The maximum value between right and left carotid c-IMT was considered for calculations. Two physicians were responsible for carrying out the carotid ultrasonographic examinations. The intraobserver coefficients of variation were, respectively, 1.2 and 1.1%; the between-observer coefficient of variation was 2.9%.

Ultrasonography of the abdomen was performed using a 3.5 MHz convex probe (Sonoline G50; Siemens, Germany) by a single trained physician for diagnosing the presence of abdominal aorta (AA) aneurysm, liver steatosis, and gallbladder stones. AA aneurysm was diagnosed, according to recent guidelines [25], as the presence of focal dilatation of AA >3 cm. Liver steatosis was defined as the presence of at least one of the following: bright liver echo pattern (fine, packed, and high-amplitude echoes with consequent brightness of the liver), increased liver-kidney contrast, possible evidence of vascular blurring, and deep attenuation signs [26]. Gallstone screening was done according to current guidelines [27].

Fasting plasma glucose (FPG), total cholesterol, high-density lipoprotein (HDL) cholesterol, triglycerides (Tg), uric acid, and creatinine concentrations were ascertained using standard clinical chemistry methods (Glucosio HK UV, Colesterolo tot. Mod P/D, Colesterolo HDL gen 3 mod P/917, Trigliceridi, Acido urico MOD P/917, and Creatininaenzimatica; Roche Diagnostics, Monza, Italy). Basal insulin concentrations (Elecsysinsulina; Roche Diagnostics, Monza, Italy), high sensitivity C-reactive protein (hs-CRP; B-analyst hsCRP; Menarini diagnostics, Florence, Italy), and glycated hemoglobin (HbA1c; B-analyst HbA1c; Menarini diagnostics, Florence, Italy) were also measured. Low-density lipoprotein (LDL) cholesterol serum concentration was calculated with Friedewald's formula [28], glomerular filtration rate (eGFR) was estimated based on the CKD-EPI equation [29], and the HOMA-IR was calculated as described by Matthews et al. [30].

Data are reported as means ± SD for continuous variables and as percentages for categorical variables. Normal distribution of values of continuous variables of interest was tested using the Shapiro-Wilk test. The Student's *t*-test for independent samples was used to compare continuous variables between 2 groups, while the *χ*^2^ test was used to compare categorical variables. Participants were classified into 4 groups according to body size and metabolic health (MH-N, MUH-N, MH-O, and MUH-O). In the case of continuous variables, ANOVA evaluated differences in means between groups, and Tukey's post hoc test was used for comparison between groups. If significant differences were found for variables such as age, gender, smoking habits, and sedentary lifestyle, they were accounted for. Therefore, differences in categorical variables in the 4 groups were analyzed using the *χ*^2^ test, with stratification for age, gender, smoking habits, and sedentary lifestyle when significantly different. Similarly, if significantly different between groups, these variables were accounted for when evaluating differences between the 4 groups in continuous variables of interest (HOMA-IR, hs-CRP, eGFR, ABI, and c-IMT) using ANCOVA. In addition, their corrected values were calculated and presented as mean ± SEM. Pearson correlation coefficients were calculated to explore the associations among continuous variables. A two-tailed *P* value of <0.05 was considered significant. All analyses were done using Systat (Windows version 13.0; San Jose, CA, USA).

## 3. Results

**Figure 1:**
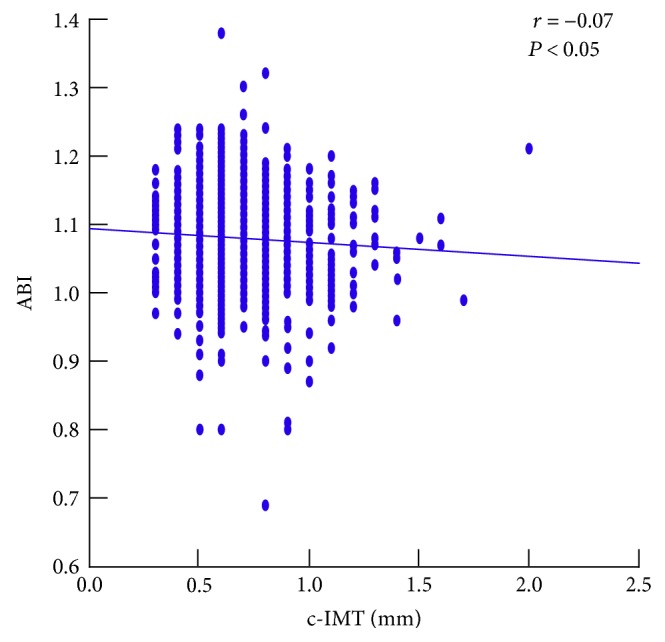
Correlation between the ankle-brachial index (ABI) and carotid intima-media thickness (c-IMT) in the cohort.

A total of 1033 participants (415 males and 618 females) were selected. Among them, 12 participants were excluded because of incomplete data and 2 participants were excluded because of pregnancy. Finally, a total of 1019 participants (408 males and 611 females) were evaluated. The prevalence of overweight was 40.4%; that of obesity was 30.7%. The physical, biochemical, and clinical characteristics of participants divided into 2 groups according to the presence of overweight-obesity are reported in Table 3. Continuous variables presented in Table 3 were normally distributed. Of the overweight-obese participants, 27.4% were MH; 36.7% of the normal-weight participants were MUH. The characteristics of the cohort divided into 4 groups according to the presence of overweight-obesity and metabolic health (MH-N, MUH-N, MH-O, and MUH-O) are reported in Table 4. The MUH subgroups (both the N and the O) exhibited higher age and prevalences of male gender and sedentary lifestyle. In Table 4, the prevalences of coronary heart disease (CHD), AA aneurism, gallbladder stones, and liver steatosis are reported, which were stratified for age, gender, and sedentary lifestyle. As reported in Table 5, age-, gender-, and sedentary lifestyle-corrected values of HOMA-IR, hs-CRP, and c-IMT were significantly different among the 4 subgroups, and higher values were observed in the MUH-N and MUH-O subgroups; no differences were observed for eGFR-, creatinine- (*P* = 0.54), and ABI-corrected values. The values of ABI and c-IMT were inversely correlated (Figure 1).

## 4. Discussion

This study investigated a general population cohort in whom the overall prevalence of overweight and obesity was very high (about 70%). This finding is in agreement with that expected in the adult population living in Sicily, in the south of Italy [31]. Compared with the normal-weight group, the overweight-obese group was older and with a higher prevalence of male gender; it also included more sedentary people and fewer smokers. With the exception of the prevalence of AA aneurysm and ABI values, which were not different between the normal-weight and overweight-obese groups, the overweight-obese group exhibited a significantly higher prevalence of comorbidities, higher values of c-IMT, and worse blood concentrations of all variables considered in this study concerning metabolism, kidney function, and inflammation. Therefore, we stratified the cohort in 4 subgroups on the basis of BMI class and MH or MUH conditions. There is a lack of consensus on the criteria to define MH, and, in general, those used in different studies were based on different combinations of the components of the metabolic syndrome. Consequently, based on the diagnostic criteria used, large differences have been reported concerning the prevalence of MH-O, ranging from 3 to 57% of obese patients [32, 33]. Probably due to the very stringent criteria adopted for defining MH (presence of no more than 1 abnormality) in our study, also including serum levels of cholesterol, as suggested by Karelis et al. [34], we observed that about 70% (corresponding to a prevalence of about 51.6% in the entire cohort) of the overweight-obese participants were MUH, a percentage that is similar to that observed in larger cohorts [35]. We also found that about 35% of normal-weight participants (about 10% of the cohort) were MUH too. As expected, older age, male gender, and a sedentary lifestyle characterized the MUH groups. Our findings are in agreement with those of one study [36], but in contrast with those of others. Phillips et al. found that lower physical activity was not associated with MUH condition, even though the questionnaires for describing the physical activity level were similar to those used in our study. They also used different criteria to stratify their cohort [37]. After stratification for age, gender, and sedentary lifestyle, we observed that MUH condition was associated with higher prevalence of coronary heart disease and AA aneurysm. The prevalence of AA aneurysm was higher in the MUH-N than in the MUH-O group. In fact, abdominal fat may induce atherosclerosis even in AA individuals [38], but it is probably a mechanical protective factor for the AA wall [39]. We also found that prevalences of gallbladder stones and liver steatosis were associated with body size category and metabolic health, even after stratifying for age, gender, and sedentary lifestyle. Gallbladder stones are frequently associated with diabetes [40], while liver steatosis, apart from being a condition frequently associated with diabetes, is considered to have an important role in promoting atherosclerosis itself [41]. Insulin resistance, as suggested by corrected HOMA-IR values, was higher in association with both overweight-obesity and MUH condition; a similar trend was also observed for hs-CRP blood concentrations. Apart from possible genetic influences that were not investigated in this study, insulin resistance can be significantly promoted by both inflammation and sedentary lifestyle and, accordingly, was associated with MUH condition in our study. In agreement with other studies, the absence of significant inflammation, even in the low-grade range, may be deemed as a characterizing trait of MH condition [42–44], thus favoring a low metabolic and cardiovascular risk profile. We confirm the importance of central body fat distribution as a factor associated with metabolic and cardiovascular complications [45]. In fact, like the MUH-O group that exhibited BMI and waist circumference values in accordance with a more pronounced degree of central obesity, the MUH-N group had uniquely higher values of waist circumference with respect to the MH-N group. Interestingly, the prevalence of cardiovascular diseases (coronary heart disease and AA aneurism) was higher in the MUH-N group than in the MH-O group. This result is in agreement with that of a recent report on a large population-based cohort from NHANES III [46], which found higher total and cardiovascular mortality in people with normal BMI and central distribution of fat than in BMI-defined obese people, particularly in the absence of central fat distribution. In agreement with these results, the value of corrected c-IMT was lower in MH groups than in MUH people, and the MUH-N group had a higher value of c-IMT than the MH-O group. Current guidelines [47] recommend the measurement of ABI for stratifying the cardiovascular risk and the screening of peripheral arterial disease. Though we found a significant correlation between ABI and c-IMT, in this study, the value of ABI was not significantly different among any of the groups considered. A possible explanation is that the ABI value is compromised for advanced stages of arterial damage, thus not an adequate indicator of vascular involvement in the general population, while it is probably a more reliable indicator in subgroups of people at higher risk [48]. Taken as a whole, these data may suggest that cardiovascular measures were associated with metabolic health independent of BMI categories, while overweight-obesity associated with MUH condition had an adjunctive unfavorable effect on metabolic measures. Finally, the phase angle is a bioelectrical body characteristic that is related to the extra-/intracellular water ratio, an interesting measure that is associated with nutritional state and good health [49]; however, we found no significant differences of the phase angle value between the 4 groups considered in this study, thus suggesting that at least the nutritional state was comparable.

Our study has the merit of presenting as completely as possible the MUH condition, in both normal-weight and overweight-obese people. Also, rigorous criteria for defining MH condition in a general population cohort were adopted, and many different metabolic and cardiovascular values were considered. In particular, with respect to MH participants, the MUH-O participants were older and more frequently of male gender and had higher degrees of central obesity, a more sedentary lifestyle, insulin resistance, and liver steatosis. Despite the low risk profile, we cannot affirm that MH-O has a benign prognosis and an attitude of caution towards this condition is merited. In fact, recent studies suggest that MH-O is a transient state [50, 51], so that it needs to be treated like MUH-O, as no clear evidence exists that MH condition has a favorable prognosis in the long run in terms of evolution towards diabetes [9, 52] and cardiovascular events [53, 54]. However, more extensive longitudinal studies are needed to clarify this.

Our study has some important limitations. The cross-sectional design did not allow us to establish any cause-effect relationship. Though we investigated a cohort whose characteristics resembled those of the general population, the modality of cohort recruitment did not allow us to affirm that our cohort was fully representative of the general population.

In conclusion, our study may contribute to improving the diagnosis of obesity in terms of metabolic health and to identifying those patients with higher metabolic and cardiovascular risk who need more in-depth strategies of treatment. In addition, we found that a significant number of normal-weight people are MUH; therefore, preventive strategies at the population level should also include an appropriate identification of this high-risk subgroup of people. Longitudinal studies on large cohorts of general populations will help to definitively clarify the real clinical importance of classifying people based on metabolic health.

## Figures and Tables

**Table 1:** Demographic characteristics of the ABCD cohort compared with those of the general population living in Palermo.

	General population of Palermo in 2011^a^			ABCD cohort in 2011			*P* ^b^	ABCD cohort in 2015			*P* ^c^
	19–59 years	60–90 years	Total	18–59 years	60–90 years	Total		18–59 years	60–90 years	Total	
Class of age	357443 (70.3%)	150700 (29.7%)	508143	843 (68.9%)	381 (31.1%)	1224	0.28	676 (65.3%)	360 (34.7%)	1036	0.07
Gender							<0.001				0.21
Male	172593 (34.0%)	64267 (12.6%)	236860 (46.6%)	263 (21.4%)	197 (16.1%)	460 (37.6%)		234	182	416 (40.2)	
Female	184850 (36.4%)	86433 (17.0%)	271283 (53.4%)	580 (47.4%)	184 (15.1%)	764 (62.4%)		442	178	620 (59.8)	
Marital status							<0.001				0.46
Single	133031 (26.2%)	13552 (2.7%)	146583 (28.9%)	191 (15.8%)	21 (1.7%)	212 (17.5%)		169 (16.3%)	14 (1.4%)	183 (17.7%)	
Married	213080 (41.9%)	95755 (18.8%)	308855 (60.0%)	595 (49.3%)	306 (25.3%)	901 (74.6%)		471 (45.5%)	301 (29.1%)	772 (74.5%)	
Divorced	6719 (1.3%)	3305 (0.7%)	10024 (2.0%)	12 (1.0%)	35 (2.9%)	47 (3.9%)		25 (2.4%)	13 (1.3%)	38 (3.7%)	
Widow/er	4613 (1.0%)	38068 (7.4%)	42681 (8.4%)	11 (0.9%)	31 (2.6%)	42 (3.5%)		11 (1.1%)	32 (3.1%)	43 (4.1%)	
Education^b^							<0.001				0.39
0 years	5972 (1.0%)	7693 (1.3%)	13666 (2.3%)	2 (0.2%)	6 (0.5%)	8 (0.7%)		1 (0.1%)	1 (0.1%)	2 (0.2%)	
0–5 years	64988 (11.3%)	89067 (15.5%)	154055 (26.8%)	56 (4.6%)	112 (9.3%)	168 (13.9%)		9 (0.9%)	87 (8.4%)	96 (9.3%)	
6–8 years	100878 (17.5%)	92354 (16.0%)	193232 (33.5%)	302 (25.0%)	122 (10.1%)	424 (35.1%)		206 (19.9%)	115 (11.1%)	321 (31%)	
9–13 years	75285 (13.1%)	79443 (13.8%)	154728 (26.9%)	354 (29.3%)	92 (7.6%)	446 (36.9%)		326 (31.5%)	107 (10.3%)	433 (41.8%)	
>13 years	29429 (5.1%)	30306 (5.3%)	59735 (10.4%)	117 (9.7%)	44 (3.7%)	161 (13.4%)		134 (12.9%)	50 (4.8%)	184 (17.8%)	
Unemployed^d^			22000 (4.3%)			58 (4.8%)	0.07			56 (5.4%)	0.47

*P* values were obtained with the *χ*^2^ test; ^a^http://www.tuttitalia.it/sicilia/81-palermo/statistiche/popolazione-eta-sesso-stato-civile-2015/ (accessed on December 30, 2015); ^b^ABCD cohort in 2011 versus general population of Palermo in 2011; ^c^ABCD cohort in 2015 versus 2011; ^d^data relative to general population of Palermo (range of age 19–90 years) in 2001 (http://www.comune.palermo.it/opendata_dld.php?id=105 accessed on 30 December 2015).

**Table 2:** List of physically demanding jobs.

Jobs considered medium HPA level (M)	Jobs considered high HPA level (H)
Housewife (<65 years) with children	Mechanic
	Truck driver
	Welder
	Varnisher
	Tinsmith
	Laborer
	Carpenter
	Painter
	Plumber
	Bricklayer
	Woodworker
	Baker
	Metalworker
	Farmer
	Docker

HPA = habitual physical activity.

**Table 3:** Physical and biochemical characteristics of the cohort divided according to the presence of overweight-obesity.

	Normal-weight (BMI < 25 kg/m^2^)	Overweight-obesity (BMI ≥ 25 kg/m^2^)	*P* ^a^
*n* (%)	294 (28.9)	725 (71.1)	
Male (%)	32.0	43.3	<0.001
Age	45 ± 15	55 ± 13	<0.001
Smokers (%)	21.4	14.5	0.01
Physical inactivity (%)	55.6	62.4	0.04
Body weight (kg)	60.4 ± 8.2	81.1 ± 14.9	<0.001
BMI (kg/m^2^)	22.7 ± 1.7	30.7 ± 4.8	<0.001
Waist circumference (cm)	82.9 ± 7.5	102.2 ± 11.3	<0.001
Phase angle BIA (°)	6.8 ± 1.1	6.9 ± 2.8	0.29
Type 2 diabetes (%)	2.7	10.5	<0.001
Hypertension (%)	16.0	41.3	<0.001
CHD (%)	1.0	5.4	<0.001
AA aneurysm (%)	1.7	1.5	0.83
Cholelithiasis (%)	7.8	13.8	0.01
Hepatic steatosis (%)	25.9	53.0	<0.001
Blood concentrations			
hs-CRP (mg/dl)	0.16 ± 0.28	0.29 ± 0.44	<0.001
Cholesterol (mg/dl)	199 ± 40	207 ± 41	0.005
LDL cholesterol (mg/dl)	118 ± 38	127 ± 36	<0.001
HDL cholesterol (mg/dl)	66 ± 17	58 ± 16	<0.001
Triglycerides (mg/dl)	81 ± 46	108 ± 58	<0.001
Uric acid (mg/dl)	4.4 ± 1.1	5.1 ± 1.3	<0.001
Glucose (mg/dl)	86 ± 12	95 ± 22	<0.001
Insulin (mU/ml)	6.75 ± 3.39	12.1 ± 9.16	<0.001
HbA_1_c (%)	5.3 ± 0.5	5.6 ± 0.7	<0.001
HOMA-IR	1.46 ± 0.91	2.95 ± 3.05	<0.001
eGFR (ml/min/1.73 m^2^)	99.8 ± 16.9	90.6 ± 17.4	<0.001
ABI	1.08 ± 0.07	1.08 ± 0.07	0.21
c-IMT max (mm)	0.61 ± 0.21	0.72 ± 0.22	<0.001

Mean ± SD or percentages. ^a^Student's *t*-test for independent samples, *χ*^2^ test if appropriate; CHD: coronary heart disease; AA: abdominal aorta; ABI: ankle-brachial index; BIA: bioelectrical impedance analysis; BMI: body mass index; c-IMT max: maximum carotid intima-media thickness; eGFR: estimated glomerular filtration rate; HbA1c: glycated hemoglobin; HDL: high-density lipoproteins; HOMA-IR: homeostasis model assessment of insulin resistance; hs-CRP: high-sensitivity C-reactive protein; LDL: low-density lipoproteins.

**Table 4:** Physical and biochemical characteristics of the cohort divided according to the presence of overweight-obesity and diabetes-prediabetes, hypertension, and hypertriglyceridemia or low HDL cholesterol (healthy = 0-1 conditions; unhealthy = at least 2 conditions).

	Normal-weight		Overweight-obesity		*P* ^a^
	Healthy (*n* = 187)	Unhealthy (*n* = 97)	Healthy (*n* = 202)	Unhealthy (*n* = 530)	
Male (%)	24.6	45.4	30.2	48.5	<0.001
Age	39 ± 12^b,c,d^	57 ± 12^c^	47 ± 13^b,d^	59 ± 11	<0.001
Smokers (%)	20.9	21.6	12.9	15.2	0.07
Physical inactivity (%)	51.6	63.9	49.0	67.3	<0.001
Body weight (kg)	60.1 ± 7.9^c,d^	60.3 ± 8.5^c,d^	70.6 ± 14.2^d^	82.7 ± 14.9	<0.001
BMI (kg/m^2^)	22.4 ± 1.7^c,d^	23.2 ± 1.4^c,d^	29.1 ± 4.6^d^	31.2 ± 4.8	<0.001
Waist circumference (cm)	80.9 ± 6.9^b,c,d^	86.5 ± 7.1^c,d^	96.8 ± 10.7^d^	104.0 ± 11.1	<0.001
Phase angle BIA (°)	6.8 ± 1.1	6.6 ± 1.3	6.8 ± 1.1	6.9 ± 3.2	0.72
Prevalence of (%)					
CHD	0	3.1	1.0	7.0	<0.001
AA aneurysm	0	4.2	0	2.3	<0.001
Cholelithiasis	4.8	13.4	10.5	15.1	0.01
Hepatic steatosis	25.1	28.1	40.5	57.2	<0.001
Insulin (mU/ml)	6.38 ± 3.15	7.51 ± 3.66	9.13 ± 5.57	13.9 ± 9.97	0.13
HOMA-IR	1.33 ± 0.79^d^	1.74 ± 1.08^d^	1.94 ± 1.26^d^	3.32 ± 3.41	<0.001
hs-CRP (mg/dl)	0.15 ± 0.30^d^	0.18 ± 0.25	0.26 ± 0.42	0.30 ± 0.45	<0.001
eGFR (ml/min/1.73 m^2^)	105.3 ± 14.6^b,c,d^	89.4 ± 16.6^c^	99.7 ± 16.9^d^	87.3 ± 16.3	<0.001
ABI	1.08 ± 0.07	1.07 ± 0.07	1.09 ± 0.06	1.08 ± 0.07	0.05
c-IMT max (mm)	0.54 ± 0.13^b,c,d^	0.74 ± 0.27^c^	0.62 ± 0.19^d^	0.76 ± 0.21	<0.001

Mean ± SD or percentages. ^a^ANOVA or *χ*^2^ test (stratified by age, gender, and physical inactivity) if appropriate. *P* < 0.05 versus ^b^normal-weight unhealthy, ^c^obese healthy, and ^d^obese unhealthy; CHD: coronary heart disease; AA: abdominal aorta; ABI: ankle-brachial index; BIA: bioelectrical impedance analysis; BMI: body mass index; c-IMT max: maximum carotid intima-media thickness; eGFR: estimated glomerular filtration rate; HbA1c: glycated hemoglobin; HDL: high-density lipoproteins; HOMA-IR: homeostasis model assessment of insulin resistance; hs-CRP: high-sensitivity C-reactive protein; LDL: low-density lipoproteins.

**Table 5:** Estimated means of the dependent variables corrected for the covariates age, gender, and physical inactivity.

	Normal-weight		Overweight-obesity		*P* ^a^
	Healthy (*n* = 187)	Unhealthy (*n* = 97)	Healthy (*n* = 202)	Unhealthy (*n* = 530)	
HOMA-IR	1.39 ± 0.22	1.72 ± 0.28	1.97 ± 0.19	3.29 ± 0.12	<0.001
hs-CRP (mg/dl)	0.12 ± 0.04	0.19 ± 0.05	0.25 ± 0.03	0.32 ± 0.02	0.006
eGFR (ml/min/1.73 m^2^)	94.1 ± 1.1	93.1 ± 1.3	94.5 ± 0.9	92.7 ± 0.5	0.08
ABI	1.08 ± 0.01	1.07 ± 0.01	1.09 ± 0.01	1.08 ± 0.01	0.56
c-IMT max (mm)	0.66 ± 0.01	0.70 ± 0.02	0.67 ± 0.01	0.70 ± 0.01	<0.001

Mean ± SD; ^a^ANCOVA; ABI: ankle-brachial index; c-IMT max: maximum carotid intima-media thickness; eGFR: estimated glomerular filtration rate; HOMA-IR: homeostasis model assessment of insulin resistance; hs-CRP: high-sensitivity C-reactive protein.
